# Growth differentiation factor 10 induces angiogenesis to promote wound healing in rats with diabetic foot ulcers by activating TGF-β1/Smad3 signaling pathway

**DOI:** 10.3389/fendo.2022.1013018

**Published:** 2023-01-13

**Authors:** Qingsong Zhao, Jinmei Xu, Xu Han, Zheqi Zhang, Jiahui Qu, Zhifeng Cheng

**Affiliations:** Department of Endocrinology, The Fourth Affiliated Hospital of Harbin Medical University, Harbin, China

**Keywords:** GDF-10, TGF-β1/Smad3 signaling, diabetic foot ulcer, wound healing, angiogenesis, GEO database

## Abstract

**Background:**

Diabetic foot ulcer (DFU) represents a highly-prevalent complication of diabetes mellitus (DM). Herein, the current study sought to identify the role of growth differentiation factor 10 (GDF-10) in wound healing in DFU *via* regulation of the transforming growth factor-beta 1 (TGF-β1)/Smad3 pathway.

**Methods:**

DM- and DFU-related microarray datasets GSE29221 and GSE134431 were firstly retrieved, and weighted gene co-expression network analysis (WGCNA) was carried out to construct a co-expression network affecting wound healing in DFU, followed by differential analysis. A protein-protein interaction (PPI) network of the DFU-related genes was subsequently constructed, and the core genes and signaling pathways in DFU were screened with the Gene Ontology and Kyoto Encyclopedia of Genes and Genomes functional analyses. A DFU rat model was constructed for mechanism verification of the effect of GDF-10 on wound healing in DFU.

**Results:**

WGCNA screened five co-expression modules, and the brown module was most closely-related to DM. Clustering analysis screened 4417 candidate genes, of which 175 differential genes were associated with wound healing, further involved in TGF-β1/Smad3 signaling pathway regulation of wound healing in DFU. The PPI network analysis predicted that GDF-10 might regulate the TGF-β1/Smad3 signaling pathway to participate in DFU development. Results of animal experimentation showed that the wound healing rates of NFU, DFU, DFU + GDF and GDF + SIS3 groups on the 22nd day were (87.66 ± 6.80)%, (56.31 ± 7.29)%, (71.64 ± 9.43)% and (55.09 ± 7.13)%, respectively. Besides, the expression of TGF-β1 in NFU, DFU, DFU + GDF and GDF + SIS3 groups was 0.988 ± 0.086, 0.297 ± 0.036, 0.447 ± 0.044, and 0.240 ± 0.050, respectively, and that of Smad3 was 1.009 ± 0.137, 0.145 ± 0.017, 0.368 ± 0.048, and 0.200 ± 0.028, respectively. Specifically, GDF-10 exerted a significant diminishing effect on fasting blood glucose level, and promoted wound healing in DFU rats, in addition to up-regulation of VEGF, FGF, Ang-1, TGF-β1, Smad3 and enhancement of IL-1b, IL-6, TNF-a and MMP-9, thereby promoting fibroblast proliferation, collagen deposition and angiogenesis.

**Conclusions:**

Our findings highlight that GDF-10 may promote angiogenesis by activating TGF-β1/Smad3 signaling, thereby promoting wound healing in DFU rats.

## Introduction

Diabetic foot ulcer (DFU) is understood as a consequence of peripheral neuropathy and peripheral arterial disorder in patients afflicted by diabetes mellitus (DM) (1). As a refractory complication of diabetes, DFU is featured by disturbed inflammatory, as well as impaired proliferative stages of wound healing (formation of granulation tissue, collagen deposition, proliferation of fibroblast cells and angiogenesis as hallmarks) (2). DFU accounts for significant morbidity and mortality, and can result in hospitalization and lower limb amputation if not treated in a timely-manner (3). In light of the same, it is prudent to promote diabetic wound healing for prevention of ulcer infections as well as subsequent amputations (4). Interestingly, a number of growth factors are closely-correlated with the repair cells, and further possess great potentials in wound repair, among which fibroblast growth factor is one of them (5). Meanwhile, activation of the PI3K/Akt/mTOR pathway can lead to the up-regulation of VEGF, FGF and EGF expressions, facilitating cell growth and migration, angiogenesis and collagen synthesis, inducing EMT and accelerating wound healing (6).

Growth differentiation factor 10 (GDF-10), also known as bone morphogenic protein-3b (Bmp3b), is a member of the transforming growth factor (TGF)-β superfamily (7), and intriguingly, possess the ability to promote wound healing in diabetic patients (8). In addition, GDF-10 is well-known for its important roles in maintaining homeostasis and glucose metabolism, and even diminishing cardiovascular risk (9). What’s more, GDF-10 has been shown to protect against islet graft rejection in recipient diabetic mice (10). Herein, initial analysis using the Gene Expression Omnibus (GEO) database predicted that GDF-10 and TGF-β1/Smad3 signaling pathway might participate in wound healing in DFU. As previously reported, the up-regulation of GDF-10 contributes to the selective activation of Smad3 phosphorylation, which is dependent on the TGF-β receptor (11). Inherently, TGF-β1 is regarded as a multifunctional gene with regulatory functions in scar formation for wound healing and angiogenesis (12). Importantly, the secretion of TGF-β1 is crucial for angiogenesis in the process of wound healing in DFU (13). Meanwhile, Smad proteins serve as key downstream regulators of the signaling activities regulated by TGF-β family, including TGF-βs and GDF members (14). Furthermore, a prior study indicated that up-regulation of TGF-β1 and Smad3 expression by lncRNA H19 can aid the promotion of wound healing of DFU by regulating the biological behaviors of fibroblasts (15). Taking the aforementioned reports into consideration, we proposed a hypothesis that GDF-10 might play a regulatory role in wound healing in DFU with the involvement of the TGF-β1/Smad3 signaling pathway, and thus performed a series of experiments for verification.

## Materials and methods

### Ethical approval

The current study was performed with the approval of the Ethics Committee of The Fourth Affiliated Hospital of Harbin Medical University. All animal experimentation was conducted in strict accordance with the guidelines for the care and use of laboratory animals.

### GEO microarray dataset acquisition

Firstly, the DM expression profile dataset GSE29221 was retrieved from the GEO database, which included 12 non-DM tissue samples and 12 DM tissue samples. This database was based on the platform information GPL6947. Additionally, the DFU dataset GSE134431 was obtained, comprising of 8 normal samples and 6 DFU samples, and was based on the platform information GPL18573.

### Weighted gene co-expression network analysis

A co-expression network of DM samples was constructed using the WGCNA algorithm. Gene co-expression network was established with the R software “WGCNA” package, and differentially expressed genes (DEGs) were screened and utilized to construct a weighted co-expression network. Hierarchical clustering analysis was subsequently performed using the Hclust function, then appropriate soft threshold was selected using the “pickSoftThreshold” function, and adjacent matrix was converted into topological overlap matrix. Hierarchical clustering dendrogram was constructed, and the genes with similar expression were divided into different modules and 50 genes were selected as the minimum number of genes in the module. To merge possibly similar modules, 0.25 was defined as the threshold of cutting height. Finally, the expression profile of each gene was summarized by the module genes, the correlation between the module genes and traits was calculated, and the most relevant modules were selected for further analysis.

### Differential analysis

Differential analyses were carried out using the R software “limma” package. DEGs in GSE29221 and GSE134431 microarray datasets were screened with logFC > 1 and *p <* 0.05 serving as the screening condition. Volcano maps and expression heatmaps of DEGs were drawn simultaneously employing the “ggplot2” and “pheatmap” packages.

### Intersection gene screening and functional enrichment analysis

Candidate genes were obtained through the intersection of the key genes in the weighted gene co-expression network and the DEGs in the GSE29221 and GSE134431 microarray datasets using the R software “venn” package. Protein-protein interaction (PPI) analysis based on the intersection genes was performed with “species” set as “human”. The PPI results were subsequently ranked by gene correlation with the R software. Functional enrichment analysis was performed on the selected DEGs using the SangerBox website.

### DM model establishment

A total of 48 Wistar rats (aged 6-weeks-old, weighing 200-220 g) were purchased from Beijing Vital River (Beijing, China), and reared in a specific-pathogen-free animal room (temperature: 22-25°C, humidity: 60-65%) under 12-h light/dark cycles. The included rats were allowed to acclimatize for one week prior to experimentation.

The rats were randomly divided into the two following groups: the control group (n = 12, rats were fed with standard diet) and the DM group (n = 36, rats were fed with high fat and high glucose diet). Prior to model establishment, the rats were fasted for 12 h with *ad libitum* access to water. The rats were weighed on the day of model establishment, blood samples were collected from the tail vein, and basal blood glucose levels were assessed with a glucose meter (Omnitest plus, Melsungen, Germany). Next, streptozocin (STZ; V900890, Merck, New Jersey, USA) was dissolved in a pH4.2 0.1 mol/L citric acid-sodium citrate solution (Aladdin, Shanghai, China) to prepare 1% solution. A single intraperitoneal injection of STZ solution at a dosage of 55 mg/kg body weight was administered to induce DM, while an intraperitoneal injection of an equal volume of citric acid-sodium citrate solution was performed for rats in the control group. After 72 h of STZ injection, the fasting blood glucose levels were measured with blood from the tail vein. Rats presenting with blood glucose level higher than 16.7 mmol/L, and typical symptoms of polydipsia, increased appetite, polyuria and wasting were regarded as DM rats. Rat body weight and blood glucose were measured weekly, and insulin was injected to achieve blood glucose control at 20 – 25 mmol/L.

### DFU model establishment and grouping

After maintenance of hyperglycemia in DM rats (n = 40) for 8 weeks, the rats were treated with ketamine (dosage of 75 mg/kg, i.p.) and toluene thiazide (dosage of 10 mg/kg, i.p.) (X1251, Merck, New Jersey, USA) for anesthesia. A rectangular-shaped wound was created on each rat’s instep using a soft clear plastic template, and a layer of skin with a standard area of 2 mm × 5 mm was then removed. The DM rats were randomly divided into the following different experimental groups (n = 10): the DFU group (DM rats injected with same volume of double-distilled water), the DFU + GDF-10 group (DM rats injected with 0.1 mg/kg of GDF-10 recombinant protein [1543-BP-025/CF, R&D Systems, Minneapolis, MN] *via* rat femoral artery and intermuscular of ischemic tissue around the wound), and the DFU + GDF-10 + SIS3 group (DM rats injected with 0.1 mg/kg of GDF-10 recombinant protein and 2 μM Smad inhibitor SIS3 (S3552, Selleck, Houston, Texas, USA) *via* rat femoral artery and intermuscular of ischemic tissue around the wound). Another group of rats without DM induction was also subjected to wound injury to serve as control rats.

All rats were administered their respective concoctions at 1, 3, 5, 7, 10, 14, and 21 days, and the wound area was photographed using a camera. After 22 days, the rats were anesthetized with ether to obtain blood samples, followed by euthanasia, and the wound tissue was quickly removed and placed at -80°C for preservation.

### Measurement of the wound area

The wound area was recorded with a camera at 1, 4, 6, 8, 11, 15, and 22 days, and calculated using the ImageJ analysis software. Calculation of wound healing percentage: wound healing percentage = [(initial wound area - wound area at calculation day)/initial wound area] * 100.

### Hematoxylin and eosin staining

Fresh wound tissues of rats were collected, fixed with 4% paraformaldehyde, fixed with xylene, embedded with paraffin, and sliced. Following hydration, the slices were stained with hematoxylin (C0105, Beyotime, Shanghai, China) for 5-10 min and with eosin for 30 s - 2 min, followed by immersion in 70% ethanol for 10 s, 80% ethanol for 10 s, 90% ethanol for 10 s, absolute ethanol for 10 s. Afterwards, the slices were cleared twice with xylene, each for 5 min, sealed with neutral gum, and visualized under a microscope.

### Masson trichrome staining

Fresh wound tissue of rats was collected, fixed with 4% formaldehyde solution, dehydrated, embedded, and sectioned. The paraffin-embedded sections were dewaxed with xylene and stained with Weigert hematoxylin for 10 min. After washing, the sections were soaked in Lichunred for 8 min, 2% glacial acetic acid for 1 min, 1% molybdate acid for 4 min, aniline blue for 5 min, then 0.2% glacial acetic acid-acid solution for 2 min, and treated with 95% alcohol. Later, the sections were cleared with xylene and finally sealed with neutral gum. The Masson-stained sections were visualized using a microscope.

### Immunofluorescence

Fresh wound tissue of rats was fixed with 4% paraformaldehyde solution, dehydrated, embedded, and sectioned. Next, the sections were incubated with CD31 antibody (ab24590, Abcam, Cambridge, MA, USA) and SMA (ab128107, 1:100, Abcam, Cambridge, MA, USA) at 4°C overnight, and then treated with goat anti-mouse IgG H&L secondary antibody (ab150116, 1:200, Abcam, Cambridge, MA, USA) or goat anti-rabbit IgG H&L (Alexa Fluor^®^ 488, ab150077, 1:200, Abcam, Cambridge, MA, USA) for 90 min at room temperature. Subsequently, the nuclei were stained with DAPI (D9542, Merck, New Jersey, USA), and the tissue sections were visualized under a fluorescence microscope (ApoTome 2, Carl Zeiss MicroImaging, Inc., Thornwood, NY, USA). Vascular number was determined by counting three random regions around the wound using the ImageJ analysis software.

### Reverse transcription-quantitative polymerase chain reaction

Total RNA content was isolated from fresh wound granulation tissue of rats, homogenized using the TRIzol reagent (10296010, Thermo Fisher Scientific, Rockford, IL, USA) (1 mL per 100 mg tissue), and then mixed with chloroform (200 μL) and centrifuged at 4°C and 12000 g for 10 min. Next, the upper aqueous phase was collected, and mixed with isopropanol (500 μL) to precipitate RNA. Subsequently, the RNA was dissolved in RNA enzyme-free water (10-30 μL) and quantified by Nanodrop (Nanodrop 3300, Thermo Fisher Scientific, Rockford, IL, USA). One μg of total RNA was reverse-transcribed with the TaqMan reverse transcription reagent (N8080234, Thermo Fisher Scientific, Rockford, IL, USA). PCR analysis was performed using the PowerUp SYBR Green premix kit (A25741, Thermo Fisher Scientific, Rockford, IL, USA). Primer sequences are illustrated in **Supplementary Table 1**.

### Enzyme-linked immunosorbent assay

Granulation tissue from fresh wounds of rats was collected, homogenized with pre-cooled PBS, centrifuged at 4°C at 1000 rpm for 20 min, followed by collection of supernatant. The levels of tumor necrosis factor-α (TNF-α) (H052-1, NanJing JianCheng Bioengineering Institute, Nanjing, China), interleukin-6 (IL-6) (H007-1-1, NanJing JianCheng Bioengineering Institute, Nanjing, China), interleukin- (IL)-1β (H002, NanJing JianCheng Bioengineering Institute, Nanjing, China), matrix metalloproteinase-9 (MMP-9) (E-EL-R3021, 7.81-500 ng/mL, Elabscience, TX, USA), TGF-β1 (70-EK981-96, 31.25-2000 pg/mL, Multi sciences (Lianke Biotech, Co., LTD., Hangzhou, China), vascular endothelial growth factor (VEGF) (ab100786, 0.82-200 pg/mL, Abcam, Cambridge, MA, USA), angiopoietin-1 (Ang1) (E-EL-R0626c, 0.16-10 ng/mL, Elabscience, TX, USA) were determined as per kit instructions.

### Statistical analysis

All data were analyzed using the SPSS 21.0 statistical software (IBM Corp. Armonk, NY). Measurement data were expressed as mean ± standard deviation. Data comparisons between two groups were performed using *t*-tests. Data comparisons among multiple groups were conducted by one-way analysis of variance. A value of *p <* 0.05 was regarded statistically significant.

## Results

### WGCNA screened five co-expression modules of genes associated with DM

DM is a highly-prevalent metabolic disease characterized by hyperglycemia, with poor control inducing severe complications, of which DFU represents one of the most common chronic complications in patients with DM (16). Herein, we adopted the WGCNA algorithm to analyze the DFU-related genes, and functional enrichment analysis was further performed to clarify the biological functions of core genes. An online database was utilized to construct a PPI network to further investigate the mechanism of core genes in DFU.

**Figure 1:**
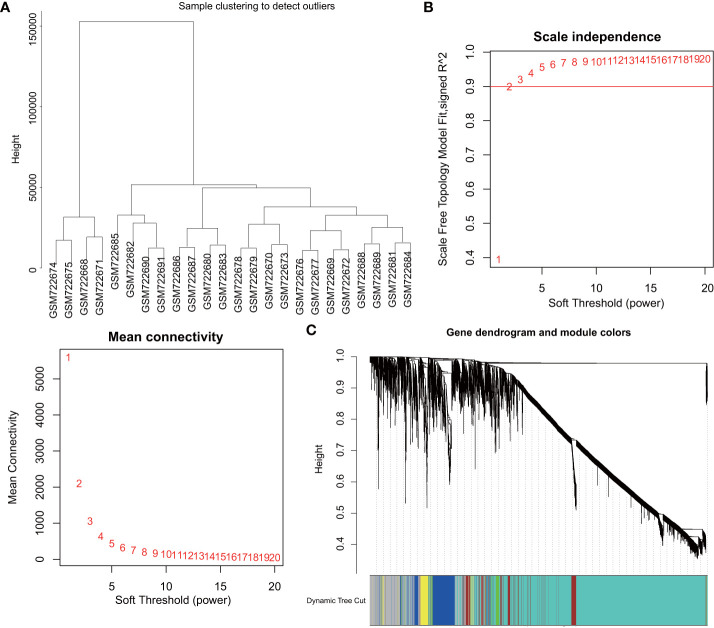
WGCNA identifies DM-related gene co-expression modules. **(A)**, Clustering dendrogram of GSE29221 gene expression profile. **(B)**, Scale-free fit index (top) of various soft threshold β and average connectivity (below); correlation coefficient (0.9) is represented by red line. **(C)**, Clustering dendrogram of gene expression network constructed by WGCNA; each color represents a module in the gene co-expression network.

Firstly, marker genes associated with DM were identified with the construction of a scale-free co-expression network with the R software package “WGCNA”. The DM related GSE29221 microarray was selected for cluster evaluation, which comprised of 12 control samples and 12 DM samples. To test sample outliers, the samples were hierarchically clustered according to the distribution of sample expression values (**Figure 1A**), and the samples included in the analysis were slightly different. β = 20 (scale-free R^2^ = 0.9) was regarded as the soft threshold to establish a scale-free network and establish a gene expression network (**Figure 1B**). Five co-expression modules were identified in the DM expression profile, with each color representing a different module (**Figure 1C**).

### Clustering analysis identified 4417 candidate genes related to DFU

**Figure 2:**
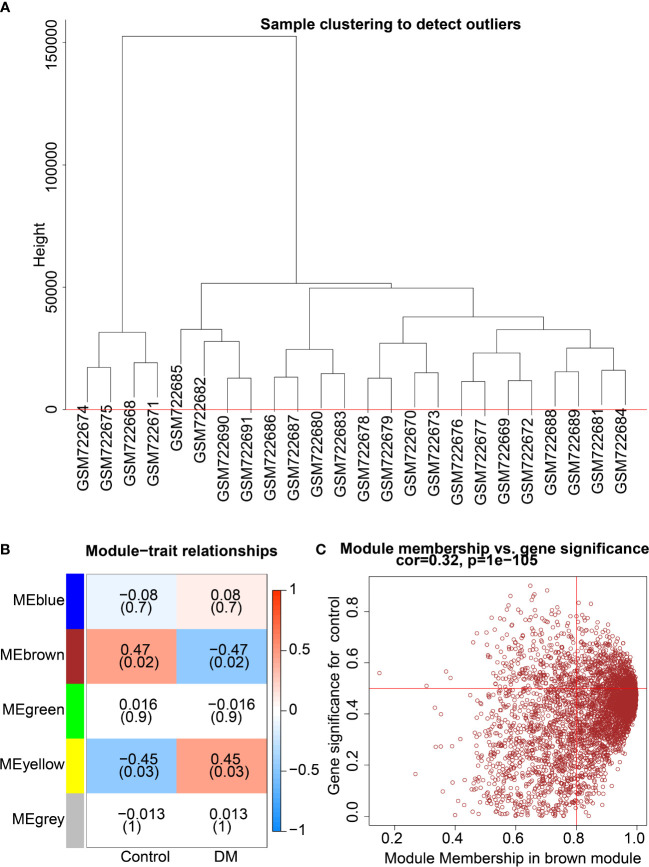
Screening of key co-expression module for DM-related genes. **(A)**, Clustering analysis according to correlation between modules to obtain expression cluster analysis diagram between modules. **(B)**, Correlation heat map between modules and traits in DM; each cell contains corresponding correlation and *p* value. **(C)**, Module correlation diagram of the brown module.

All genes were assessed and clustered by their correlation to obtain an expression cluster analysis map between the individual modules (**Figure 2A**). Pearson correlation analysis was carried out for each module gene with different groups. Five co-expression modules were obtained for subsequent analysis (**Figure 2B**), including the Meblue module (containing 621 genes), Mebrown module (containing 4417 genes), Megreen module (containing 156 genes), Meyellow module (containing 215 genes) and Megrey module (containing 877 genes) (**Figure 2C**; **Supplementary Figures 1A–D**). Module feature correlation analysis showed that the Mebrown module (containing 4417 genes) was most associated with the disease, and the correlation coefficient was 0.47. Therefore, the 4417 genes in the brown module were regarded as DM related genes.

### Differential analysis selected 175 DEGs associated with wound healing in DFU

**Figure 3:**
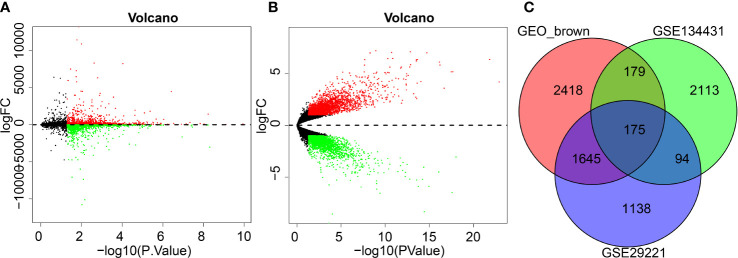
GEO differential analysis for screening DFU-related DEGs. **(A)**, Volcano map of GSE29221 dataset (Black color represents genes not differently expressed, red color represents up-regulated genes and green color represents downregulated genes). **(B)**, Volcano map of GSE134431 dataset (Black color represents genes not differently expressed, red color represents up-regulated genes and green color represents downregulated genes). **(C)**, Venn diagram shows brown modules obtained from intersection of DEGs in GSE134431 and GSE29221 datasets.

To further screen the key genes implicated in DFU, differential analyses were performed on the GSE29221 dataset and a total of 5338 DEGs were selected, of which 2286 were up-regulated and 3052 were down-regulated (**Figure 3A**; **Supplementary Figure 2A**).

Following differential analyses of the DFU-related microarray dataset GSE134431, a total of 5522 DEGs were obtained, of which 2961 genes were up-regulated and 2561 genes were down-regulated (**Figure 3B**; **Supplementary Figure 2B**).

To further screen the key genes involved in DFU, the DEGs of the GSE134431 microarray dataset, the DEGs of the GSE29221 microarray dataset and the brown module genes were intersected, which reared 175 intersection genes for subsequent functional analysis (**Figure 3C**).

### The TGF-β1/Smad3 signaling pathway might be involved in wound healing in DFU

**Figure 4:**
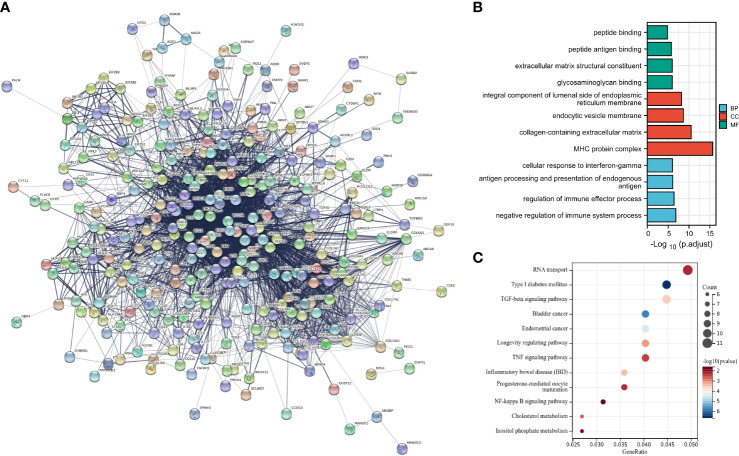
Construction of DFU-related gene network and screening of key pathway. **(A)**, PPI network of DFU related genes. **(B)**, GO function analysis of candidate genes at the biological process, cellular component and molecular function levels. **(C)**, KEGG pathway enrichment analysis of candidate genes.

Decreased angiogenesis in damaged skin is regarded as a key reason for the impaired wound healing in DM complications (17). Accordingly, angiogenesis-related genes were screened using the GSEA database, and a total of 132 genes were obtained. The STRING analysis was adopted to construct a PPI network map of the angiogenesis and DFU-related genes (**Figure 4A**). In regard to previous studies, the molecular functions of the candidate gene regulation were deeply explored, and the GO function analysis and KEGG pathway analysis of the candidate genes were carried out.

Results of GO function illustrated that candidate genes in biological process (BP) were primarily enriched in the negative regulation of immune system process, regulation of immune effector process, antigen processing, presentation of endogenous antigen and cellular response to interferon-gamma in biological processes. Moreover, candidate genes in cellular component (CC) were primarily enriched in the MHC protein complex, collagen-containing extracellular matrix, endocytic vesicle membrane, integral component of lumenal side of endoplasmic reticulum membrane in cellular components. Additionally, candidate genes in molecular function (MF) were largely enriched in the glycosaminoglycan binding, extracellular matrix structural constituent, peptide antigen binding and peptide binding in molecular function (**Figure 4B**).

Furthermore, results of KEGG pathway analysis showed that the candidate genes were primarily enriched in RNA transport, type 1 DM, TFG-β signaling pathway, bladder cancer, endometrial cancer, longevity regulating pathway, TNF signaling pathway and inflammatory bowel disease, progesterone-mediated oocyte maturation, NF-kappa B signaling pathway, cholesterol metabolism and inostiol phosphate metabolism (**Figure 4C**). Among the aforementioned, type 1 DM and TGF-β signaling pathway were predominated.

Together, the above mentioned findings indicated that the candidate genes largely exerted roles in biological processes including angiogenesis regulation, extracellular matrix organization, extracellular structural organization, and the candidate genes were enriched in the endoplasmic reticulum cavity, collagen trimers, and collagen-containing extracellular matrix.

Moreover, the KEGG analysis results demonstrated that the candidate genes were involved in RNA transport, type 1 DM, TFG-β signaling pathway, TNF signaling pathway, and NF-κB signaling pathway, among which the TFG-β signaling pathway was predominated.

### GDF-10 might be involved in wound healing in DFU *via* regulation of the TGF-β1/Smad3 signaling pathway

**Figure 5:**
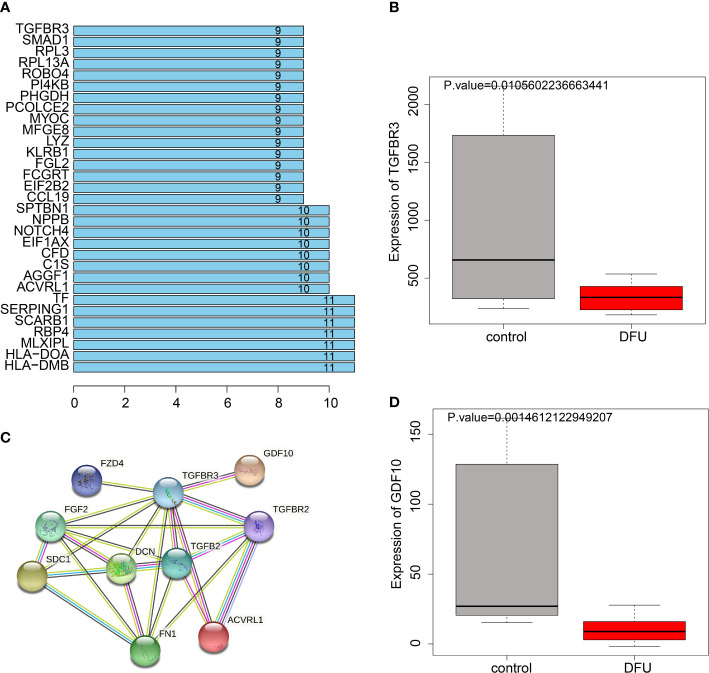
PPI relationship between TGFBR3 and GDF10. **(A)**, Degree values of the genes. **(B)**, PPI diagram showing interaction of genes with TGFBR3. **(C)**, Expression of TGFBR3 in DFU in GSE134431 dataset. **(D)**, GDF10 expression in DFU in GSE134431 dataset.

The interaction network between DEGs and angiogenesis-related proteins was constructed using a PPI network, and TGFBR3 was interacted with more proteins (**Figure 5A**). TGFBR3 represents a type III TGF-β superfamily receptor transmembrane, which influences angiogenesis and migration by regulating the TGF-β1/Smad3 signaling pathway (18). Further PPI analysis of the TGFBR3-related genes showed that TGFBR3 interacted with GDF-10 (**Figure 5B**), and these two genes were markedly poorly-expressed in DFU (**Figure 5C, D**). Meanwhile, BMP3B is a member of the TGF-β superfamily, and also known as GDF-10. Existing evidence suggests that GDF-10 can promote the differentiation and growth of osteoblasts (19). Meanwhile, GDF-10 can activate the TGFBR1/Smad3/ERK pathway to promote the growth and migration of tumor cells (20), and further regulate the recruitment and activation of Smad family transcription factors (21).

The transforming factor TGF-β1 is well-characterized for its role in DM (22), and further activates two key downstream mediators including Smad2 and Smad3 (23). Meanwhile, TGF-β is also implicated in the regulation of proliferation, differentiation, migration and survival of various different cell types (including blood endothelial cells) (24). Given the fact that GDF-10 is a member of the TGF-β family, we speculated that GDF-10 could activate the TGF-β1/Smad3 signaling pathway to stimulate angiogenesis, and thus promote wound healing in DFU.

### GDF-10 stimulated angiogenesis and promoted wound healing in DFU rats

**Figure 6:**
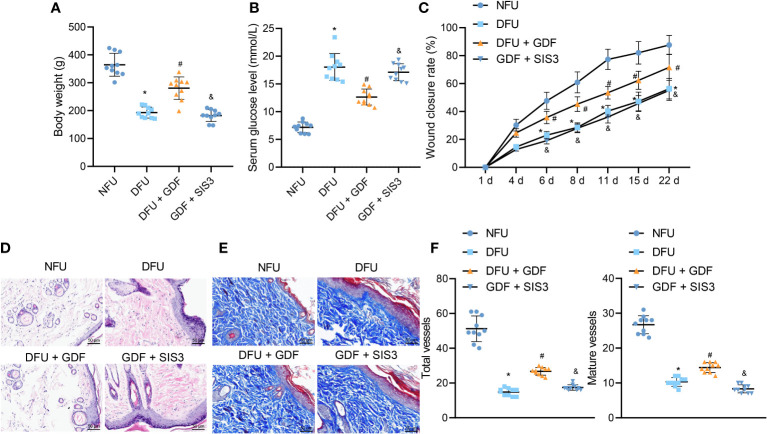
Effects of GDF10-stimulated angiogenesis on the wound healing in DFU rats. **(A)**, Rat weight. **(B)**, Blood glucose of rats after fasting. **(C)**, Representative picture of DFU rats after GDF10 treatment. **(D)**, HE staining of pathological changes in control rats and DFU rats of different treatment. **(E)**, Masson trichrome staining of collagen deposition in control rats and DFU rats of different treatment. **(F)** Immunofluorescence detection of angiogenesis in wounds: red fluorescent marks α-SMA, green fluorescent marks CD31, and blue fluorescent marks nucleus. Measurement data were expressed in the form of mean ± standard deviation. One-way ANOVA was used for comparison between multiple groups, and repeated measures ANOVA was used to compare data between groups at different times. *p < 0. 05 vs. the NFU group. ^#^ *p <*0.05 vs. the DFU group. ^&^ *p <*0.05 vs. the DFU + GDF group. Ten rats were used in each group.

DFU rat models were established to further investigate the effect of GDF-10 on wound healing in DFU rats. The weight of DM rats showed a gradual decline, while GDF-10 treatment led to the opposing trends. Compared with the DM rats treated with GDF-10, the weight of DM rats treated with GDF-10 and Smad inhibitor SIS3 was decreased (**Figure 6A**). Meanwhile, fasting glucose results showed that blood glucose was increased in DFU rats, while DFU rats treated with GDF-10 presented with decreased blood glucose. Compared with the DFU rats treated with GDF-10, the DFU rats treated with GDF-10 and SIS3 presented with increased blood glucose levels (**Figure 6B**). Moreover, the wound healing area results showed that the control rats showed faster healing, and the redness and swelling around the wound subsided 15 days later. In contrast to the control rats, poor wound healing was noted in the DFU rats, and granulation tissue grew more slowly, in dark red color; there were more inflammatory exudates around the wound, and reduced healing area. Compared to the DFU rats, the DFU rats treated with GDF-10 exhibited minimal redness around the wound and no significant exudate, accompanied by rapidly growing granulation tissue and increased healing area, and the trends of which could be reversed by further treatment of SIS3 (**Figure 6C**).

Granulation tissue formation, collagen deposition, fibroblast proliferation and vascularization are widely-adopted as markers for wound healing (2). HE staining results illustrated that control rats exhibited less inflammatory cells and few new capillaries. Meanwhile, the DFU rats presented with more neutrophils and lymphocytes and rarely new capillaries. Compared with the DFU rats treated with GDF-10, the DFU rats co-treated with GDF-10 and SIS3 exhibited reduced fibroblasts and neonatal capillaries, and increased infiltration of inflammatory cells (**Figure 6D**). Masson trichrome staining was further performed to visualize collagen deposition in tissues, the results of which demonstrated fewer collagen fibers in DFU rats. Compared with DFU rats treated with GDF-10, those treated with GDF-10 and SIS3 in combination exhibited fewer collagen fibers (**Figure 6E**). Immunofluorescence detected wound neovascularization, and the results illustrated that total and mature vessels were decreased in DUF rats relative to control rats. Compared with the DFU rats treated with GDF-10, the DFU rats treated with GDF-10 and SIS3 presented with decreased total and mature vessels (**Figure 6F**).

**Figure 7:**
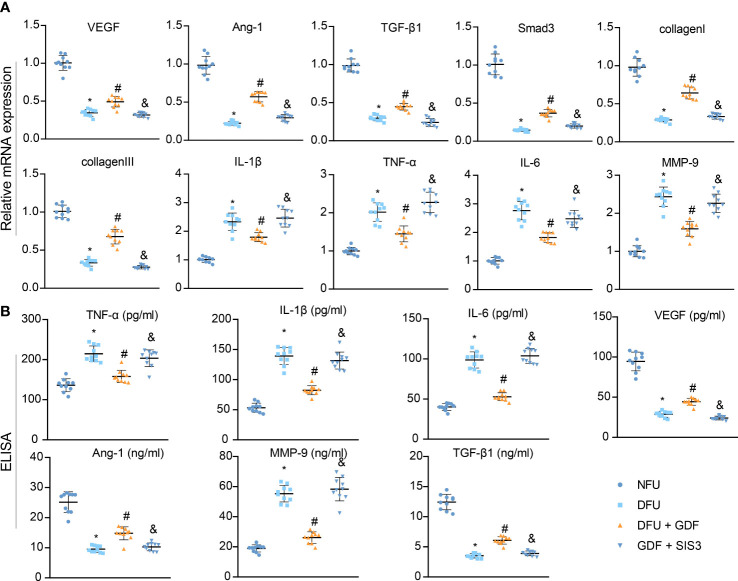
Effects of GDF10-stimulated angiogenesis on the wound healing-related factors in DFU rats. **(A)**, RT-qPCR for detecting mRNA expression of Smad3, VEGF, Ang-1, TGF-β1, collagenI, collagenIII, IL-1β, IL-6, TNF-α, and MMP-9. **(B)**, ELISA for determining expression of VEGF, Ang-1, TGF-β1, IL-1β, IL-6, TNF-α, and MMP-9. Measurement data were expressed in the form of mean ± standard deviation. One-way ANOVA was used for comparison between multiple groups, and repeated measures ANOVA was used to compare data between groups at different times. *p < 0. 05 vs. the NFU group. ^#^ *p <*0.05 vs. the DFU group. ^&^ *p <*0.05 vs. the DFU + GDF group. Ten rats were used in each group.

Furthermore, RT-qPCR results demonstrated that the expression levels of Smad3, VEGF, Ang-1, TGF-β1, collagen I, and collagen III were decreased and the expression levels of IL-1β, IL-6, TNF-α, and MMP-9 were enhanced in DFU rats, while the opposing trends were noted following GDF-10 treatment; relative to GDF-10 treatment alone, both GDF-10 and SIS3 treatment brought about reductions in the expressions of Smad3, VEGF, Ang-1, TGF-β1, collagen I, and collagen III, but elevated those of IL-1β, IL-6, TNF-α, and MMP-9 (**Figure 7A**). ELISA results further depicted that expression levels of VEGF, Ang-1 and TGF-β1 were decreased, but expression levels of IL-1β, IL-6, TNF-α and MMP-9 were elevated in DFU rats, while the opposing trends were documented following GDF-10 treatment. Relative to the DFU rats treated with GDF-10, the DFU rats treated with GDF-10 and SIS3 presented with decreased expression levels of VEGF, Ang-1, and TGF-β1 and increased expression levels of IL-1β, IL-6, TNF-α and MMP-9 (**Figure 7B**).

The above findings highlighted that GDF-10 could reduce blood glucose, TNF-α, IL-1β and IL-6 in DM rats and promote the expression of VEGF, TGF-β1 and Ang-1 to accelerate wound healing in DFU *via* activation of the TGF-β1/Smad3 signaling pathway; the therapeutic effect could be reversed after treatment with the Smad inhibitor SIS3.

## Discussion

DFU is one of the most prevalent complications of DM, exerting a burden on not only the patients, but also on overall health, nursing practice, as well as the social environment (25). Herein, the current study sought to identify the role of GDF-10 in wound healing in DFU, and the obtained findings indicated that GDF-10 could facilitate wound healing in DFU *via* the activation of the TGF-β1/Smad3 signaling pathway, underlining a novel modality for DFU treatment.

Firstly, GEO-based analyses were widely-adopted in our study. The WGCNA algorithm screened five co-expression modules, wherein the brown module was most closely-associated with DM. Subsequent clustering analysis screened 4417 candidate genes, such that a total of 175 DEGs were found to be associated with wound healing. What’s more, the TGF-β1/Smad3 signaling pathway was predicted as a key pathway for wound healing in DFU. In line with our findings, an ever-increasing number of studies have documented the roles of the TGF-β1/Smad3 signaling pathway in DFU and wound healing. For instance, the up-regulation of TGF-β and Smad2/3 by baicalin led to the suppression of wound healing in STZ-induced rat models of DFU (26). On the other hand, silencing of TGF-β1 and Smad3 *via* down-regulation of lncRNA H19 results in attenuated wound healing in DFU (15). Moreover, activation of TGF-β/Smad3 signaling pathway by fatty acid extracts can aid in facilitating cutaneous wound healing through promotion of angiogenesis (27). Further in accordance with our discovery, Zhang F et al. indicated that TGF-β1 is highly-expressed in the serum and the dorsalis pedis arteries of DFU patients, while being markedly lower in the muscles with ulcers relative to controls (28). Additionally, the activity of Smad3 has been suggested to serve as the requisite for wound healing functions of Periplaneta americana extracts (29).

Additional mechanistic experimentation in our study based on the prediction from PPI network analysis revealed that GDF-10 might promote wound healing in DFU by activating the TGF-β1/Smad3 signaling pathway to induce angiogenesis. The same was further confirmed by means of *in vivo* animal experimentation. Despite the role of GDF-10 in DFU being rarely reported, prior studies have unfolded its role in various other types of diseases. Existing evidence indicates that GDF-10 is implicated in the anti-inflammatory activity of certain cytokines, and can further relieve relieving nerve injury-induced neuropathic pain in rats (30). Moreover, another prior study reported that silencing of GDF-10 leads to neuropathic pain by activation of the N-methyl-D-aspartate receptor (31). However, up-regulation of GDF-10 by erythropoieti has previously shown to promote axonal sprouting for neurological recovery after brain injury (32). Interestingly, GDF-10 has also been demonstrated to be capable of reducing islet graft rejection in mice models of DM (10). Further in accordance with our findings, a number of authors have explored the interaction between GDF-10, Smad and TGF-β. For example, Kraunz KS et al. suggested that GDFs transduce their signals *via* the Smad protein family in a direct manner (33). Besides, another study reported that up-regulation of GDF-10 can selectively activate Smad3 phosphorylation relying on the TGF-β receptor (11). Similary, the study performed by Zhou et al. reported that specifically over-expressed GDF-10 can up-regulate SMAD7 and down-regulate p-SMAD2 expression, thereby promoting tumor cell apoptosis and inhibiting tumor cell proliferation (34). Furthermore, over-expression of GDF-10 brings about the up-regulation of p-SMAD3, consequently promoting the activation of stromal fibroblasts and the proliferation and migration of tumor cells (20). Similarly, the interaction between GDF10 and TGFBR3 regulates epithelial mesenchymal transition and tumor cell resistance through SMAD2/3 pathway in oral squamous cell carcinoma (35). A prior study has unveiled that GDF-10 through interaction with the transcription factor RUNX family transcription factor 2 can activate the TGFβRI/Smad3/ERK pathway in oral squamous cell carcinoma cells (20). In addition, fibrocytes can release TGF-β1 to diminish the accumulation of collagens while maintaining tissue integrity for cornea wound healing, and TGF-β1 activates Smad3 to down-regulate the levels of α-SMA (36). Together, the above mentioned findings and evidence highlight the promoting role of GDF-10-mediated TGF-β1/Smad3 signaling pathway in wound healing in DFU.

## Conclusions

**Figure 8:**
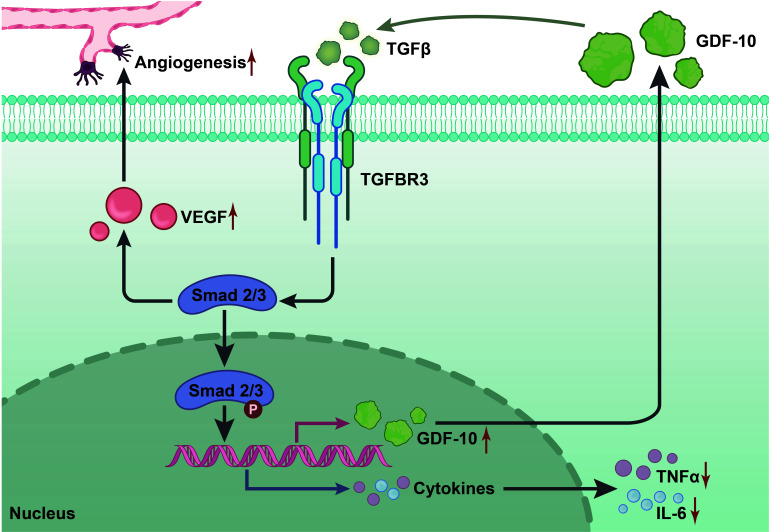
Molecular mechanism diagram of GDF10 regulating TGF-β1/smad3 signaling pathway to affect wound healing in rats with DFU. GDF10 reduces STZ-induced blood glucose levels in rats, inhibits the expression of inflammatory factors (TNF-a, IL-1β, IL-L 6) and promotes growth factors (VEGF, TGF-β1), thus activating TGF-β1/smad3 signaling to regulate the expression of Ang-1 and collagen I/collagen III to induce angiogenesis, which promotes wound healing in DFU.

To sum up, findings uncovered in the current study indicate that GDF-10 can reduce blood glucose levels in STZ-induced rats, inhibit the expression of inflammatory factors and promote that of the growth factors (VEGF, TGF-β1) to activate the TGF-β1/Smad3 signaling pathway, which induces angiogenesis, thereby promoting wound healing in DFU (**Figure 8**). Our findings may provide a novel direction in the search for diagnostic and therapeutic targets of DFU, but the results still needs further validation due to limited information regarding the role of GDF-10 in DFU.

## Data availability statement

The original contributions presented in the study are included in the article/**Supplementary Material**. Further inquiries can be directed to the corresponding author.

## Ethics statement

The animal study was reviewed and approved by the Ethics Committee of The Fourth Affiliated Hospital of Harbin Medical University.

## Author contributions

Conception and design: QZ, JX; Administrative support: QZ, ZC; Provide research materials or patients: JQ; Data collection and aggregation: ZZ; Data analysis and interpretation: XH; Manuscript writing: All Authors; Final Approval of Manuscripts: All Authors. All authors contributed to the article and approved the submitted version.
