# Supplementary material for: Growth differentiation factor 10 induces angiogenesis to promote wound healing in rats with diabetic foot ulcers by activating TGF-β1/Smad3 signaling pathway
Source: Front Endocrinol (Lausanne). 2023 Jan 13;13:1013018. doi: 10.3389/fendo.2022.1013018 (PMC9880151; doi:10.3389/fendo.2022.1013018)
Supplement: Supplementary file 1 [file DataSheet_1.zip › Supp Fig Legends.DOCX]

**Supplementary Figure Legends**

**SUPPLEMENTARY FIGURE 1** Correlation diagram of gene modules. A, Correlation diagram of yellow module. B, Correlation diagram of blue module. C, Correlation diagram of green module. D, Correlation diagram of grey module.

**SUPPLEMENTARY FIGURE 2** Heatmap of GEO differential analysis. A, Expression heatmap of GSE29221 dataset (normal = 9, DM = 9). B, Expression heatmap of GSE134431 dataset (normal = 8, DFU = 6).
