# Supplementary figures and images for: Growth differentiation factor 10 induces angiogenesis to promote wound healing in rats with diabetic foot ulcers by activating TGF-β1/Smad3 signaling pathway
Source: Front Endocrinol (Lausanne). 2023 Jan 13;13:1013018. doi: 10.3389/fendo.2022.1013018 (PMC9880151; doi:10.3389/fendo.2022.1013018)

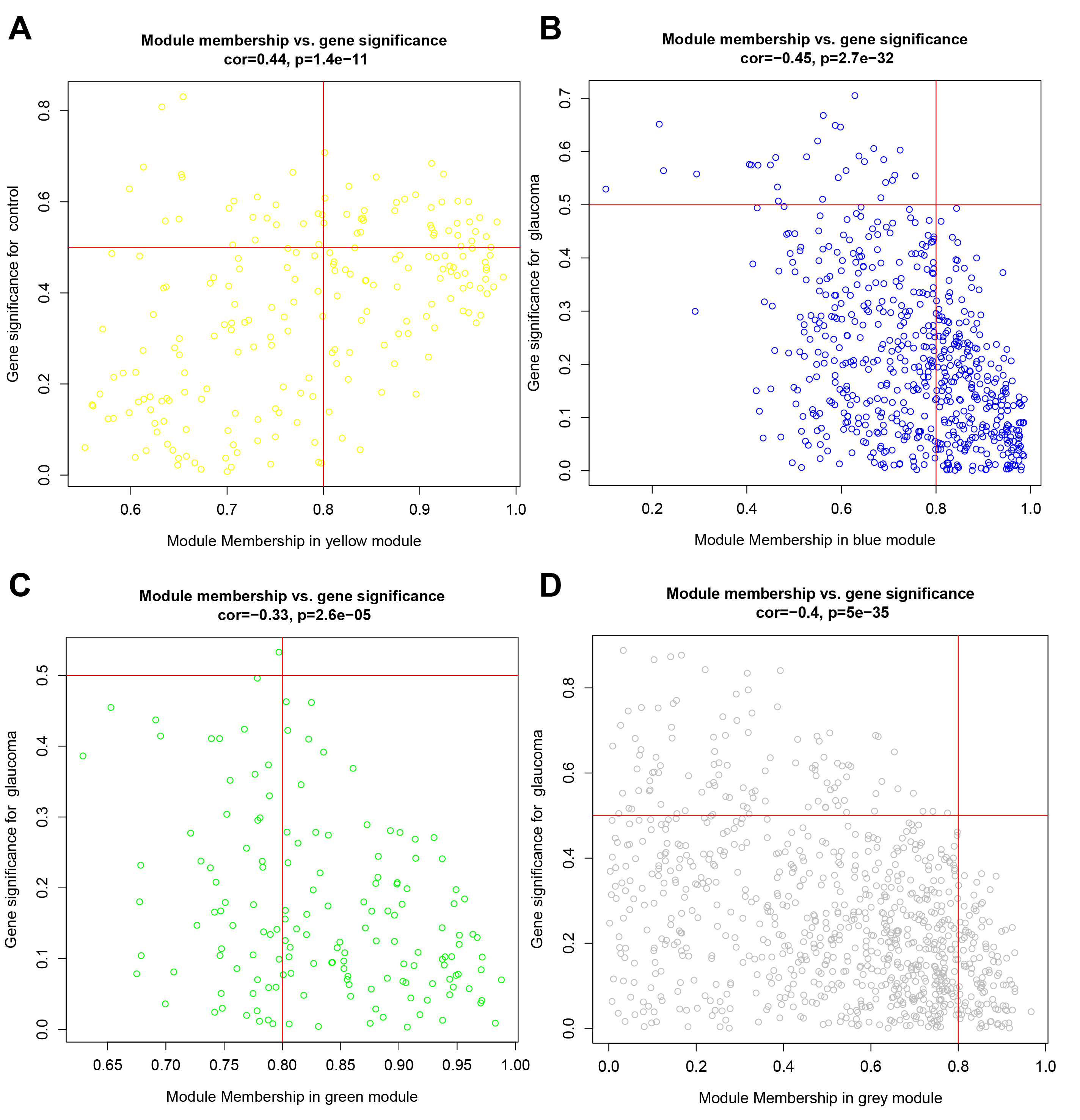

Supplement: Supplementary file 1 [file DataSheet_1.zip › Image 1.JPEG]

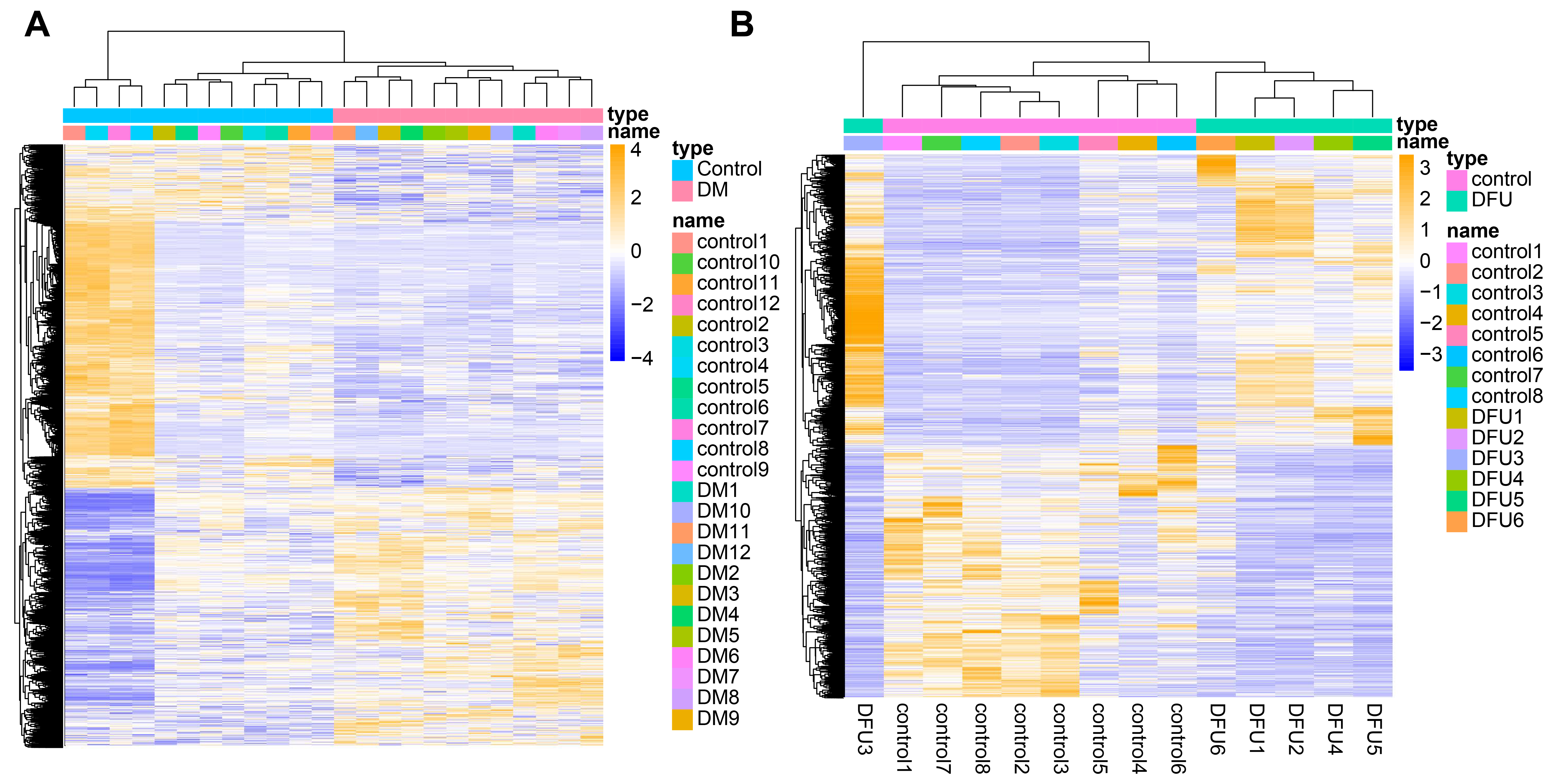

Supplement: Supplementary file 1 [file DataSheet_1.zip › Image 2.JPEG]
