# Supplementary material for: Growth differentiation factor 10 induces angiogenesis to promote wound healing in rats with diabetic foot ulcers by activating TGF-β1/Smad3 signaling pathway
Source: Front Endocrinol (Lausanne). 2023 Jan 13;13:1013018. doi: 10.3389/fendo.2022.1013018 (PMC9880151; doi:10.3389/fendo.2022.1013018)
Supplement: Supplementary file 2 [file Table_1.docx]

**Supplementary Table 1** Primer sequences for RT-qPCR

| Gene | Primer sequence |
| --- | --- |
| Smad3 (Rat ) | Forward: 5’-CAACTGCAGTGCCGCTATC-3’ |
|  | Reverse: 5’-GAAGGGCAGGATGGACGAC-3’ |
| TNF-α (Rat ) | Forward: 5’-CTCAGAGCCCCCAATCTGTG-3’ |
|  | Reverse: 5’-ACTCAGGCATCGACATTCCG-3’ |
| MMP-9 (Rat ) | Forward: 5’-GACACCACCGAGCTATCCAC-3’ |
|  | Reverse: 5’-TTTAAACGGGCTGTTTCCCCT-3’ |
| Collagen I (Rat ) | Forward: 5’-TTTGGAGAGAGCATGACCGA-3’ |
|  | Reverse: 5’-AGGGACTTCTTGAGGTTGCC-3’ |
| Collagen III (Rat ) | Forward: 5’-TGCAATGTGGGACCTGGTTT-3’ |
|  | Reverse: 5’-GGGCAGTCTAGTGGCTCATC-3’ |
| IL-1b (Rat ) | Forward: 5’-CCTATGTCTTGCCCGTGGAG-3’ |
|  | Reverse: 5’-TCAGACAGCACGAGGCATTT-3’ |
| TGF-β1 (Rat ) | Forward: 5’-GGCGGTGCTCGCTTTGTA-3’ |
|  | Reverse: 5’-TCCCGAATGTCTGACGTATTGA-3’ |
| IL-6 (Rat ) | Forward: 5’-TCATTCTGTCTCGAGCCCAC -3’ |
|  | Reverse: 5’-TGTGGGTGGTATCCTCTGTGA -3’ |
| Ang-1 (Rat ) | Forward: 5’-AGGTTGGTGGTTTGATGCCT-3’ |
|  | Reverse: 5’-CGGGAACATCCCCAGATTGT-3’ |
| VEGF (Rat ) | Forward: 5’-CGACAGAAGGGGAGCAGAAA-3’ |
|  | Reverse: 5’-GGGCTTCATCATTGCAGCAG-3’ |
| GAPDH (Rat ) | Forward: 5’-GCATCTTCTTGTGCAGTGCC-3’ |
|  | Reverse: 5’-GACTGTGCCGTTGAACTTGC-3’ |

Note: TNF-α, tumor necrosis factor-α; MMP9, matrix metalloproteinase-9; IL-, interleukin-; TGF-β1, transforming growth factor-beta 1; Ang-1, angiopoietin-1; VEGF, vascular endothelial growth factor; GAPDH, glyceraldehyde-3-phosphate dehydrogenase; RT-qPCR, reverse transcription-quantitative polymerase chain reaction.
